# MiR-221 negatively regulates innate anti-viral response

**DOI:** 10.1371/journal.pone.0200385

**Published:** 2018-08-08

**Authors:** Hongqiang Du, Shuang Cui, Yunfei Li, Guang Yang, Peiyan Wang, Erol Fikrig, Fuping You

**Affiliations:** 1 Department of Immunology, Institute of Systems Biomedicine, School of Basic Medical Sciences, Peking University Health Science Center, Beijing, China; 2 Neuroscience Research Institute; Department of Neurobiology, School of Basic Medical Sciences, Peking University, Beijing, China; 3 Department of Parasitology, Department of Public Health and Preventive Medicine, School of Medicine, Jinan University, Guangzhou, Guangdong, China; 4 Section of Infectious Diseases, Yale University School of Medicine, New Haven, Connecticut, United States of America; 5 Howard Hughes Medical Institute, Chevy Chase, Maryland, United States of America; 6 Institute of Systems Biomedicine, Department of Pathology, School of Basic Medical Sciences, Beijing Key Laboratory of Tumor Systems Biology, Peking University Health Science Center, Beijing, China; Institut de Biologie Moleculaire et Cellulaire, FRANCE

## Abstract

The innate immune system plays a critical role in the initial antiviral response. However, the timing and duration of these responses must be tightly regulated during infection to ensure appropriate immune cell activation and anti-viral defenses. Here we demonstrate that during antiviral response, a negative regulator miR-221 was also induced in an ELF4-dependent manner. We further show that ELF4 promotes miR-221 expression through direct binding to its promoter. Overexpression and knockdown assay show that miR-221 can negatively regulate IFNβ production in time of virus infection. RNA-seq analysis of miR-221 overexpressed cells revealed multiple candidate targets. Taken together, our study identified a novel negative microRNA regulator of innate antiviral response, which is dependent on ELF4.

## Introduction

Antiviral responses are initiated once the host pattern recognition receptors (PRRs), such as Toll-like receptors (TLR3, TLR7/8, TLR9) [1], cytosolic double strand DNA (dsDNA) receptors (DAI, IFI16, DDX41 and cGAS) [2–5], and RIG-I like receptors (RIG-I and Mda5) [6, 7], recognize its ligands. The ligands for RIG-I like receptors are cytoplasmic viral RNAs, and the adaptor MAVS (Mitochondrial Antiviral Signaling Protein) works downstream of these receptors and passes on the signals, leading to the transcription of NFκB and IRF3/7-dependent genes, including type I IFNs [8–11].

MicroRNAs (miRNAs) are single stranded RNAs of approximately 22 nucleotides, and are highly conserved in eukaryotes [12–14]. Mature miRNAs bind their cognate mRNAs through sequence base pair complementarity and trigger mRNA destabilization, stabilization or translational repression to modulate protein output [15, 16]. MiRNAs participate in both microbial pathogenesis and innate immunity. Previous studies showed that the complete loss of miRNAs does not impact RNA virus replication [17, 18] and that the IFN response impairs miRNA targeting [19]. For example, miR-122 plays a role in the hepatitis C virus (HCV) life cycle [20]. In addition, miRNAs may be induced by TLR and RIG-I activation in myeloid cells and act as feedback regulators of TLR and RIG-I signaling [21]. In murine macrophages, let-7e can be induced by TLR signaling, and in turn suppress TLR4 expression [22]. Moreover, vesicular stomatitis virus (VSV) infected macrophages upregulate miR-146a expression through RIG-I signaling, and miR-146a suppresses type I IFN production by targeting IRAK1, IRAK2 and TNFR-associated factor 6 sequentially, facilitating VSV replication [23].

ELF4 belongs to the ETS transcription factor family, which has at least 27 mammalian members involved in various biological processes [24]. We recently demonstrated that ELF4 regulates interferon and is critical for host defense [25]. ELF4 is activated by TBK1 (TANK Binding Kinase 1) in response to DNA and RNA virus infection, and enters the nucleus to initiate the transcription of type I IFNs [25]. In addition, ETS family members can regulate the transcription of miRNAs [26], including miR-221, which is associated with tumorigenesis [27]. We now explore the role of ELF4 in miRNA production after viral infection, and its importance in viral pathogenesis and host defense. A list of ELF4 regulatory miRNAs was generated, of which miR-221 was shown to negatively regulate antiviral innate immunity. We further show that ELF4 induce miR-221 through direct binding to its promoter. RNA-seq analysis of miR-221 overexpressed cells revealed multiple candidate targets. Our study may provide important implications for inflammatory and autoimmune diseases.

## Materials and methods

### Mice, cells and viruses

*Elf4*−/− mice on a C57BL/6J background have been described previously [28].All procedures followed the Peking University Guidelines for “Using Animals in Intramural Research” and were approved by the Animal Care and Use Committee of Peking University. iBMDM cells were a gift from Dr. Feng Shao (National Institute of Biological Sciences, Beijing). Isolation of mouse embryonic fibroblasts (MEFs) and peritoneal macrophages was performed as previously described [29]. HEK 293T were purchased from American Type Culture Collection (ATCC) and cultured in Dulbecco's modified Eagle medium (DMEM). VSV-GFP (Indiana strain) was kindly provided by Dr. John Rose (Yale University) and HSV-1 by Dr. Akiko Iwasaki (Yale University).

### Plasmids

Pcmv5-human ELF4 has been described previously [21]. The plasmids coding human STING, MAVS, TBK1, IRF3, IRF7 and STK38 have been described previously [25].

Various miR-RNAs were cloned into pCMV-MIR MicroRNA Expression Vector (OriGene).

### RNA interference

23-nt antisense sequences were designed to produce shRNA expression cassettes. Antisense sequences of classic and mutated shRNAs corresponding to positions 65–87 of miR-221(miR-221 65-AGCUACAUUGUCUGCUGGGUUUC-87).

The miR-221 specific shRNA was cloned into pGenesil-1.1 vector and the oligo sequence was as follows:

AGCTCAAAAAAGAAACCCAGCAGACAATGTAGCTCAACAGCTACATTGTCTGCTGGGTTTCTTTTTTG

Anti-has-mir-221 (inhibitor of has-mir-221) was purchased from Qiagen.

### Plaque assay and enzyme-linked immunosorbent assay

First, infect cells (~1 × 10^5^) with VSV. 24 hours later, collect supernatants and infect confluent Vero cells. Another hour later, remove supernatants and wash cells with PBS, then overlay culture medium containing 2% (wt/vol) methylcellulose upon cells for 60 hours. Next, fix cells with 0.5% (vol/vol) glutaraldehyde for 30 minutes and stain cells with 1% (wt/vol) crystal violet dissolved in 70% ethanol. Plaques were counted and viral titer as plaque-forming units per ml were calculated.

ELISA were performed using an IFN beta Human ELISA Kit (Thermo Fisher) according to manufacturer’s guide.

### Promoter reporter construction

The microRNA promoter selection was as previously described [30]. The promoter regions were designed to include all or most of the promoter sequences (about 50bps). We amplified the promoter regions from human genomic DNA, and insert them into PGL3-basic plasmid. Other promoter luciferase plasmids including *Ifnb*-luc were as described [25].

### RNA seq

HEK 293T cells were transfected with miR-221 for 6 hours. Then we harvested these cell and control cells, and purified whole RNA using RNeasy Mini Kit (Qiagen NO. 74104).The transcriptome library for sequencing was generated using VAHTSTM mRNA-seq v2Library Prep Kit for Illumina® (Vazyme Biotech Co.,Ltd, Nanjing, China) following the manufacturer's recommendations. The primary data can be accessed at Gene Expression Omnibus with the identifier”GSE107174”.

### Flow cytometry analysis

HEK293T cells, mouse embryonic fibroblasts or macrophages were infected with VSV-GFP, and harvested at the indicated time. The cells were fixed by IC fixation buffer (00-8222-49, eBioscience) and analyzed by FACS Calibur.

### Quantitative real-time PCR

Total RNA was isolated from cells using the RNeasy RNA extraction kit (Qiagen) and cDNA synthesis was performed using 1 ug of total RNA (iScriptcDNA Synthesis kit). Quantitative PCR was done with gene-specific primers and 6FAM-TAMRA (6-carboxyfluorescein–N,N,N′,N′-tetramethyl-6-carboxyrhodamine) probes or inventoried gene expression kits from Applied Biosystems (6FAM-MGB (6-carboxyfluorescein minor groove binder) probes).

### Quantitative real-time PCR of miRNA

To identify the ELF4 regulated miRNA, a selected panel of miRNA probes was purchased from Qiagen (92 miRNAs, Qiagen MIMM-105ZD-2). This kit is composed of 92 probes of the miRNAs that are related to inflammatory responses or autoimmunity and 4 internal control. Wild type or *Elf4*−/− macrophages were infected and the miRNA expression level was measured by Quantitative real-time PCR. RNA was isolated from cells using miRNeasy Mini Kit (Qiagen) cDNA synthesis was performed using 1 ug of total RNA (miScript II RT Kit miScript II RT Kit). The TaqMan probe for human miR-221 (477981_mir), mouse miR-221 (mmu481005_mir), internal control U6 (001973) were from Thermo Scientific.

### Statistical analysis

Statistical comparisons were performed with the mean ± standard error of the mean (SEM) for continuous variables. All data were statistically analyzed by unpaired 2 sample t-test with p<0.05 indicative of statistical significance. All analyses were performed using GraphPad Prism 6.

## Results

### Screening for ELF4 dependent microRNAs in response to viral infection

**Fig 1:**
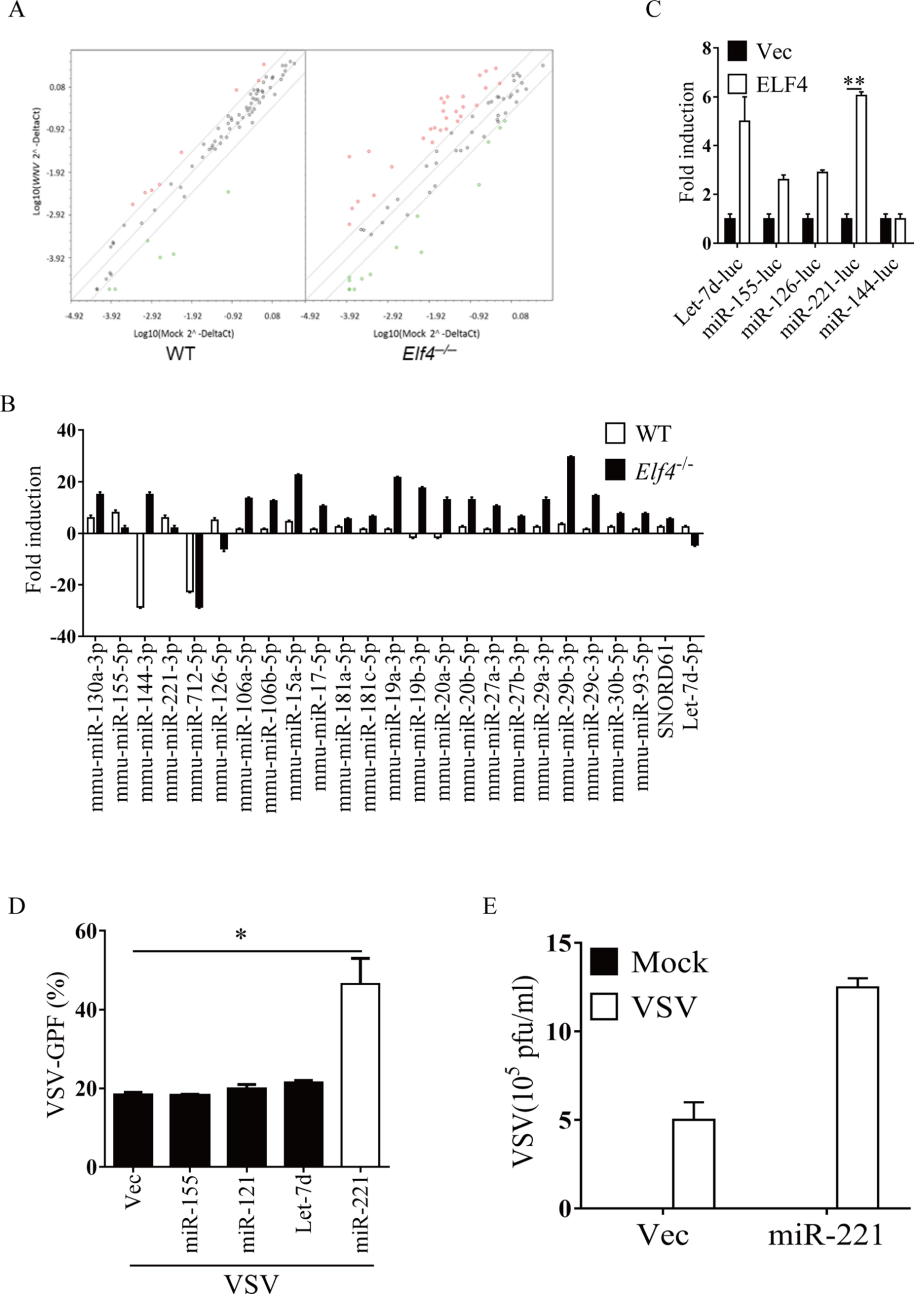
Differentially expressed microRNAs in response to viral infection of *Elf4*^*─/─*^ macrophage. (A and B) Wild type (WT) or *Elf4*^*─/─*^ peritoneal macrophages were infected with VSV. 12 hours later miRNA transcript levels were analyzed by quantitative PCR. (C) 293T cells were transfected with the indicated luciferase (Luc) plasmids, and empty vector (Vec) or ELF4. The luciferase activity of each microRNA is indicated as fold induction. (D and E) 293T cells were transfected with the indicated plasmids. 12 hours later, the cells were infected with VSV. 24 hours later the viral load (defined by percentage of GFP-positive cells) was analyzed by FACS (D) and plaque assay (E). The data are expressed as the mean ± SEM of 4 independent experiments. (n = 4 biological replicates).

We have previously found that ELF4, an ets domain transcription factor, plays a critical role in antiviral immunity [25]. A handful of miRNAs have been found to be involved in type I interferon production regulation [31]. Meanwhile, Ets1 and Ets2, both belonging to ets domain transcription factor family, were reported to regulate the expression of microRNA-126 in endothelial cells [32]. So we attempted to test whether ELF4 might regulate some miRNAs in response to viral infection. To do so, we selected 92 miRNAs, which have been shown to be involved in inflammatory responses or autoimmunity. We infected wild type and *Elf4*^─/─^macrophages with VSV and measured the expression of these miRNAs using qRT-PCR assay (Fig 1A). A small group of miRNAs were induced following VSV infection of macrophages and most of them were increased in the *Elf4*^*─/─*^macrophages compared to wild type macrophages (Fig 1B). However, the induction of miR-221, Let-7D, miR-126 and miR-155 were impaired in *Elf4*^*─/─*^macrophages (Fig 1B), suggesting that they are ELF4-dependent. To confirm that these miRNAs were regulated by ELF4, we generated luciferase reporters driven by the promoter sequence of the miRNAs. ELF4 was able to induce the reporters of miR-221, Let-7D, miR-126 and miR-155 but not miR-144 (Fig 1C). Previous study showed that miR-221 was among the most highly expressed miRNAs in macrophages [33]. We next constructed miR-221 expression plasmid and confirmed its expression (S1 Fig). We then observed that miR-221 but not the three other miRNAs could facilitate viral infection (Fig 1D and 1E). MiR-221 could therefore potentially dampen the host anti-viral immune responses.

### ELF4 directly promotes miR-221 induction

**Fig 2:**
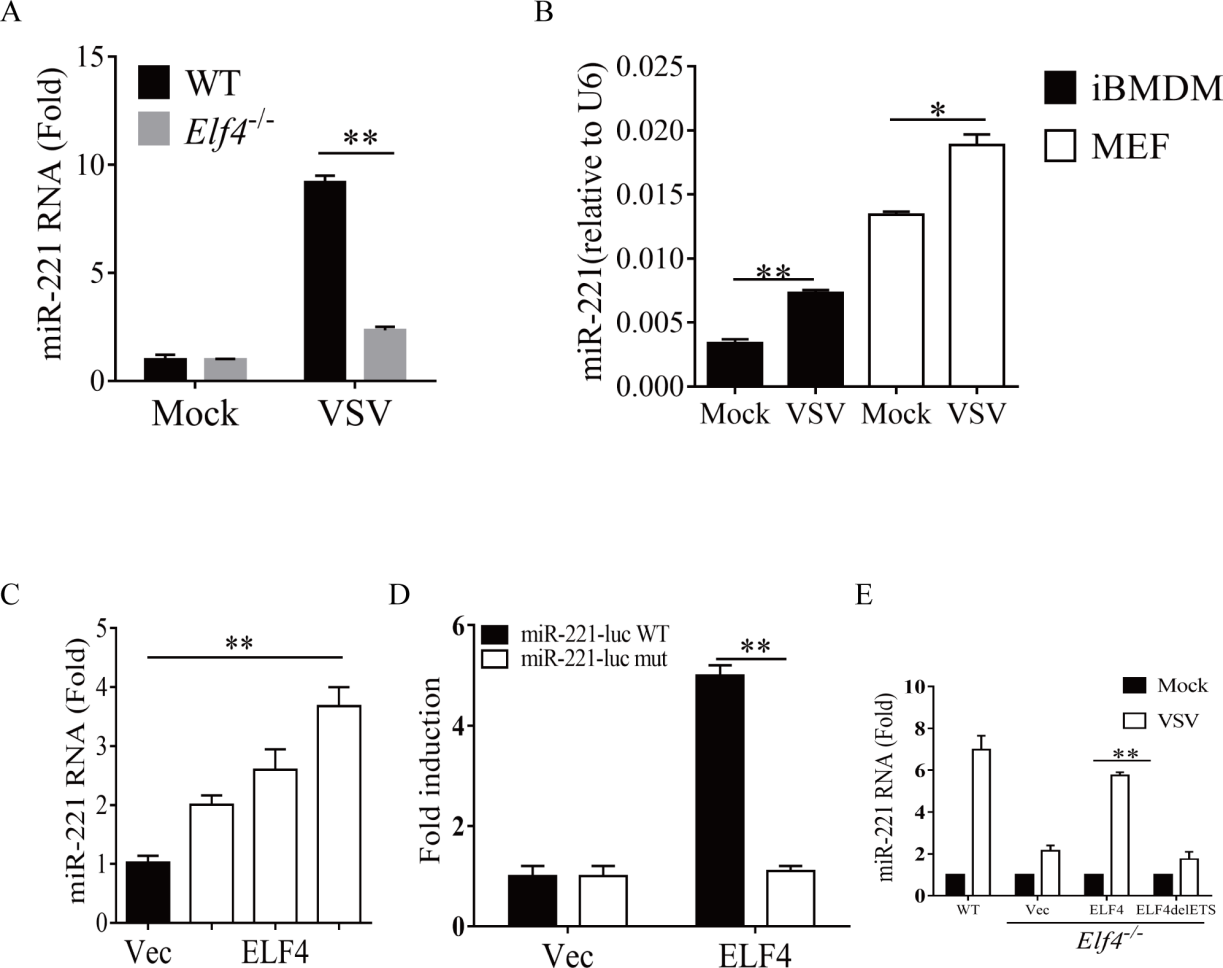
ELF4 promotes miR-221 expression through direct promoter binding. A. Wild type (WT) or *Elf4*^*─/─*^ peritoneal macrophages were infected with VSV. 12 hours later miR-221 was analyzed by quantitative PCR. (B) iBMDM or MEF cells were infected with VSV. 8 hours later, miR-221 transcripts were measured by q-PCR. (C) HEK 293T cells were transfected with empty vector (Vec) or increasing amounts of ELF4 (50ng, 100ng, 200ng). 24 hours later miR-221 transcript levels were analyzed by quantitative PCR. (D) HEK 293T cells were transfected with miR-221 promoter-driven luciferase plasmids (wild type or mutant), and empty vector (Vec) or ELF4. (E) Wild type (WT) or *Elf4*^*─/─*^iBMDM (immortalized Bone marrow derived macrophage) cells were infected with ELF4 or ELF4-del ETS lentivirus, and 24 hours later were infected with VSV. The data are expressed as the mean ± SEM of 4 independent experiments. (n = 4 biological replicates).

Before further investigation, we specifically confirmed that miR-221 is induced by VSV infection in a ELF4-dependent manner (Fig 2A) and induction of miR-221 by VSV infection is also observed in immortalized bone marrow derived macrophage and mouse embryonic fibroblast cells (Fig 2B). Next, Consistent with the reporter assay, overexpression of ELF4 triggered the production of miR-221 in a dose-dependent manner (Fig 2C). Moreover, ELF4 was not able to activate the luciferase reporter when the miR-221 promoter had a GGAA mutation—which is essential for DNA binding of ELF4 (25) (Fig 2D). It is believed that the ETS domain of ELF4 mediates the binding between ELF4 and the DNA elements. To further confirm that ELF4 promotes the transcription of miR-221 through direct DNA binding, we reconstituted full length ELF4 or the mutant lacking ETS domain into ELF4-deficient cells, followed by VSV infection. The impaired miR-221 induction was rescued by reconstituted expression of full length ELF4, but not by the mutant ELF4, in *Elf4*^─/─^ macrophages (Fig 2E). In summary, these data demonstrate that ELF4 can initiate the transcription of miR-221 in response to VSV infection.

### MiR-221 is a negative regulator of innate immune signaling

**Fig 3:**
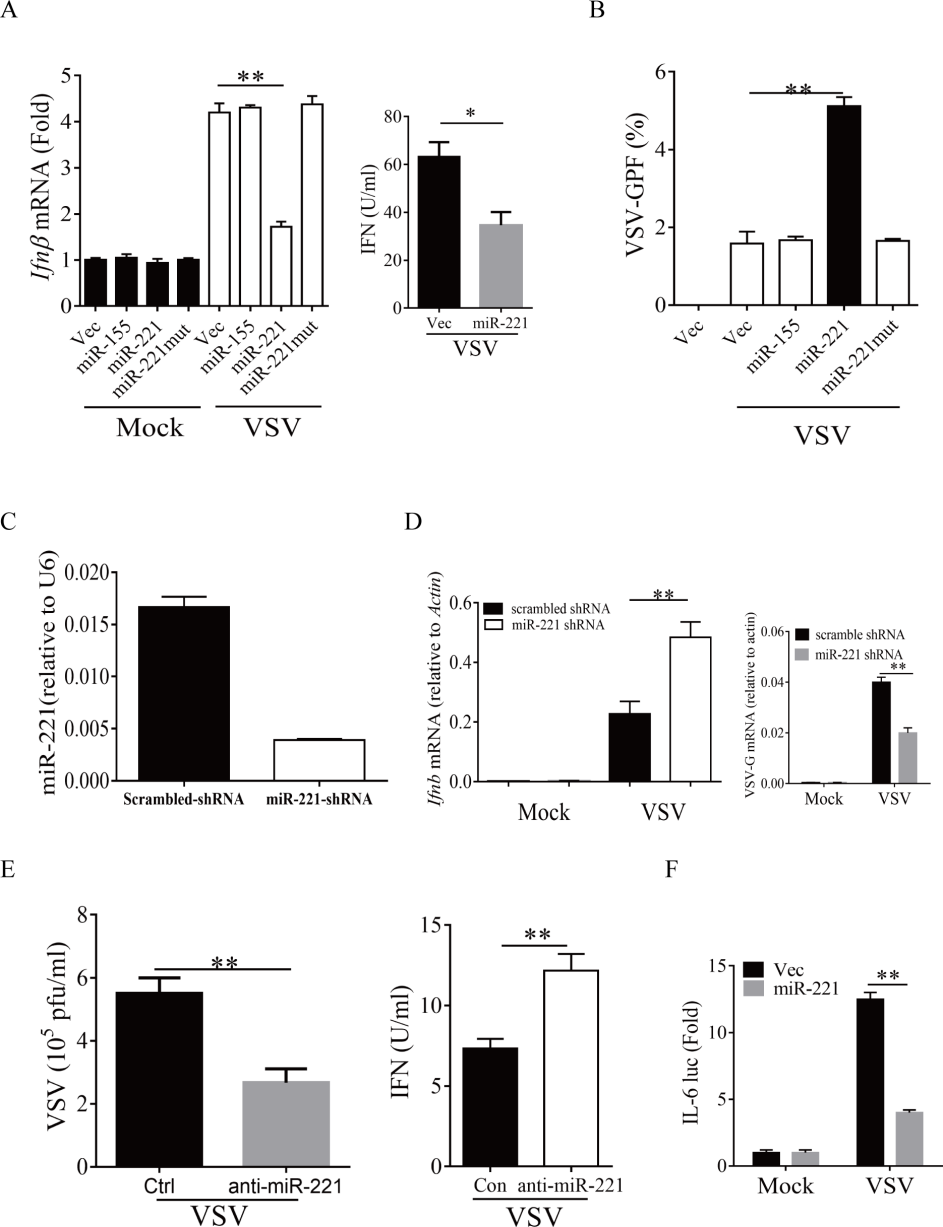
miR-221 negatively regulates innate anti-viral response. (A) HEK 293T cells were transfected with the indicated plasmids (the miR-221mut plasmid is derived from miR-221 plasmid by deleting 10 core nucleotides). 12 hours later, the cells were infected with VSV. 24 hours later IFNβ mRNA was measured by qPCR, supernatant from selected groups were subjected for ELISA. and the viral load (B) was analyzed by FACS. (C) HEK 293T cells were transfected with scrambled or miR-221 shRNA. 48 hours later, miR-221 transcripts were measured by q-PCR. (D) HEK 293T cells were transfected with the indicated plasmids, and infected with VSV at 24 hours. Another 24 hours later IFNβand VSV G protein transcript levels were analyzed by quantitative PCR. (E) HEK 293T cells were transfected with the indicated Oligonucleotides, and infected with VSV at 24 hours. Another 24 hours later supernatant were subjected to plaque assay and ELISA. (F) HEK 293T cells were transfected with the indicated luciferase (Luc) plasmids, and empty vector (Vec) or miR-221 and infected with VSV 24 hours later. As an internal control, 10 ng of pRL-TK was transfected simultaneously. The data are expressed as the mean ± SEM of 4 independent experiments. (n = 4 biological replicates).

We previously showed that ELF4 is essential for the induction of type I IFNs during host defense [25]. As ELF4 regulates the production of miR-221, we explored the role of miR-221 in anti-viral immune responses. We overexpressed miR-221 and infected the cells with VSV or herpes simplex virus-1 (HSV-1). Overexpression of miR-221 in cells inhibited the production of IFNβ (Fig 3A and S2 Fig) and facilitated viral infection (Fig 3B and S3 Fig). The miR-221 mutant with 10 core nucleotides deleted did not influence IFN induction or viral infection (Fig 3A and 3B). In line with this, the induction of IFNβ was restored, and viral replication was suppressed, when we silenced endogenous miR-221 with a specific shRNA and synthetic anti-miR (Fig 3C–3E). Inflammatory cytokines were also induced during viral infection. MiR-221 inhibited induction of IL-6(Fig 3F). Taken together, our data suggested that miR-221 is a potent negative regulator of type I IFNs production.

### MiR-221 functions downstream of TBK1 and might target multiple pathways to inhibit type I IFN production

**Fig 4:**
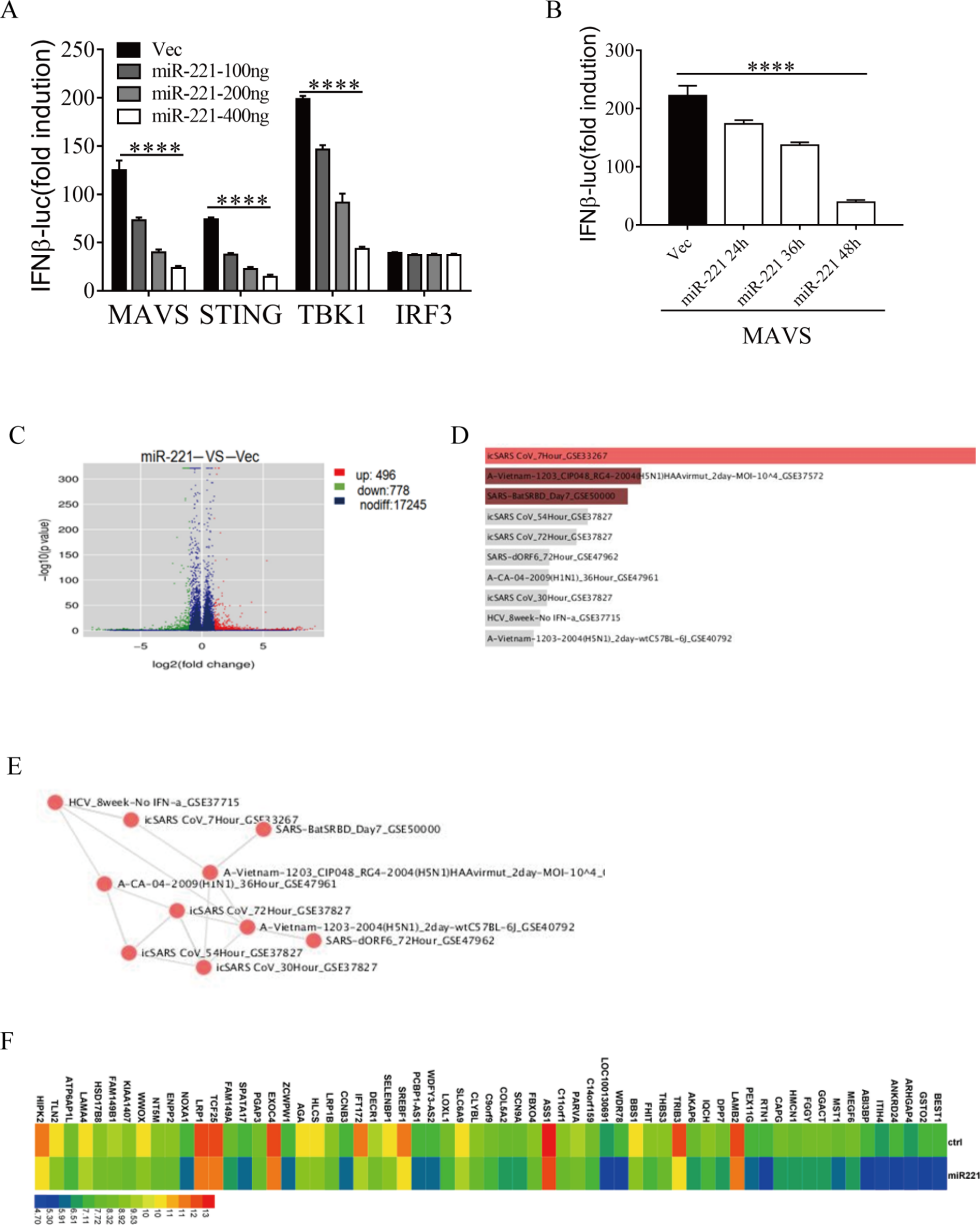
Overexpression of miR-221 downregulated genes analysis. (A) HEK 293T cells were transfected with an IFNβ-Luc plasmid and the indicated plasmids. Cells were then given miR-221 or an empty vector (Vec) control. (B) HEK 293T cells were transfected with an IFNβ-Luc plasmid, MAVS and empty vector (Vec) or miR-221. The data are expressed as the mean ± SEM of 2 independent experiments. (n = 2 biological replicates). (C) HEK 293T cells were transfected with miR-221 or empty vector (Vec). 6 hours later the cells were harvested followed by whole genome RNA-seq analysis. (D) enrichment analysis using “Virus Perturbations from GEO down” section of “Enrichr” program. (E) Network graph of downregulated genes, showing top ten clusters. (F) Heatmap of top ten clusters of downregulated genes.

Viral infection can trigger the production of type I IFNs through cytosolic nuclear acids sensors. STING (Stimulator of interferon genes) [34] and MAVS are adaptor proteins downstream of cytosolic virus sensors. Overexpression of miR-221 inhibited the induction of IFNβ by these adaptors (Fig 4A and 4B). TBK1 is a kinase downstream of MAVS and STING, and can phosphorylate and activate IRF3/IRF7. The IRF3/IRF7 complex then initiates transcription of type I IFNs. To further explore the role of miR-221, we co-transfected the cells with miR-221 and TBK1 or IRF3. MiR-221 inhibited IFNβ induction by TBK1 but not IRF3 (Fig 4A), indicating that miR-221 targets a joint signaling molecule of these two pathways, and downstream of TBK1. To further delineate the function of ELF4 and miR-221 in host defense, we used RNA-Seq to identify targets that miR-221 may use in innate immune signaling. We transfected HEK 293T cells with miR-221 or control miRNA, and then sequenced the RNAs from these cells to determine the abundance of mRNAs of the genes that were modulated by miR-221(Fig 4C). With a focus on identifying candidate positive regulators of anti-viral immunity, we analyzed the down regulated genes (miR-221 overexpression versus control, fold change>2) in the “Virus Perturbations from GEO down” section of a web-base analysis tool (http://amp.pharm.mssm.edu/Enrichr/)[35]. Many of our down regulated genes were also down regulated in multiple virus infection RNA-seq datasets (Fig 4D and 4E), such as BEST1, HIPK2 and MST1 et al (Fig 4F). It is noteworthy that the anti-virus role of these candidate genes have not been revealed. Further studies should be conducted to test their involvement in anti-virus innate immunity. Nevertheless, these virus infection related transcriptomic changes strongly indicate that miR-221 utilizes multiple pathways to inhibit anti-virus innate immunity.

## Discussion

MiRNAs are involved in the extensive network of host defense. As examples, miR-155, miR-146 and miR-21 are induced as negative regulators in a feedback mechanism to turn off TLR signaling [23, 26]. Regulation of the RLR pathway is likely somewhat similar, although the mechanisms have not yet been clearly defined [21]. We recently demonstrated that ELF4 is activated by RLR signaling after viral infection [25]. Here we revealed a role for ELF4 in miRNA production following viral infection. In particular, miR-221 is induced by VSV in an ELF4-dependent manner in different types of cells. We demonstrated that ELF4 initiated the transcription of miR-221 by binding the GGAA sequence in the promoter region of miR-221. Overexpression of miR-221 significantly inhibited the induction of IFNβ and facilitated virus infection. Therefore, miR-221 functions as a negative regulator of antiviral innate immune signaling.

In the present study, we mainly focus on the negative regulation of RLR signaling by miR-221. However, we did briefly observe that HSV-1 induced-IFNβ expression was also inhibited by miR-221 overexpression (S2 Fig), which could be responsible for the enhanced infection of HSV-1 (S3 Fig). Generally, HSV-1 activates cGAS-STING pathway to induce type I interferon and other cytokines expression, indicating miR-221 plays a broad role in regulating anti-viral response. More interestingly, both RLR and cGAS-STING pathways can also be activated by RNA or DNA species from the host cells themselves, resulting in autoimmunity or enhanced immune surveillance [36]. MiR-221 overexpression has been found in several types of tumors, and may contribute to tumor progression and therapy resistance [37]. Overexpression of type I interferons is very common in many autoimmune diseases [38] and type I interferons play important roles in tumor surveillance [39]. Thus, it is intriguing to further investigate the potentials of miR-221.

Components of innate immune signaling are often targets of miRNAs. To understand how miR-221 suppresses immune responses, RNA-seq was performed to elucidate targets of miR-221 in innate immune signaling. A handful of virus infection related down regulated genes were found, many of which are new to anti-viral pathway. Extensive work should follow these clues to fully understand the mechanism underneath the inhibition of type I IFNs production by miR-221.

## Supporting information

S1 Fig MiR-221 plasmid expressed normally. HEK 293T cells were transfected with increasing amount of miR-221 plasmid, 24 hours later, miR-221 transcripts were determined by qPCR. The data are expressed as the mean ± SEM of 2 independent experiments. (n = 2 biological replicates).
(TIF)

S2 Fig MiR-221 inhibits HSV-1 induced IFNβ expression. HEK 293T cells were transfected with an IFNβ-Luc plasmid and the indicated plasmids, 12 hours later, the cells were infected with HSV-1. Data were pooled from three independent experiments. The data are expressed as the mean ± SEM of 2 independent experiments. (n = 2 biological replicates).
(TIF)

S3 Fig MiR-221 promotes HSV-1 infection. HEK 293T cells were transfected with the indicated plasmids. 12 hours later, the cells were infected with HSV-1. 24 hours later the viral load was measured by qRT-PCR. The data are expressed as the mean ± SEM of 2 independent experiments. (n = 2 biological replicates).
(TIF)

## References

[1] Schulz O, Diebold SS, Chen M, Naslund TI, Nolte MA, Alexopoulou L, et al Toll-like receptor 3 promotes cross-priming to virus-infected cells. Nature. 2005 2 24;433(7028):887–92. 10.1038/nature03326 . 15711573

[2] Takaoka A, Wang Z, Choi MK, Yanai H, Negishi H, Ban T, et al DAI (DLM-1/ZBP1) is a cytosolic DNA sensor and an activator of innate immune response. Nature. 2007 7 26;448(7152):501–5. 10.1038/nature06013 . 17618271

[3] Unterholzner L, Keating SE, Baran M, Horan KA, Jensen SB, Sharma S, et al IFI16 is an innate immune sensor for intracellular DNA. Nature immunology. 2010 11;11(11):997–1004. 10.1038/ni.1932 . Pubmed Central PMCID: 3142795. 20890285 PMC3142795

[4] Zhang Z, Yuan B, Bao M, Lu N, Kim T, Liu YJ. The helicase DDX41 senses intracellular DNA mediated by the adaptor STING in dendritic cells. Nature immunology. 2011 9 4;12(10):959–65. 10.1038/ni.2091 . Pubmed Central PMCID: 3671854. 21892174 PMC3671854

[5] Sun L, Wu J, Du F, Chen X, Chen ZJ. Cyclic GMP-AMP synthase is a cytosolic DNA sensor that activates the type I interferon pathway. Science. 2013 2 15;339(6121):786–91. 10.1126/science.1232458 . Pubmed Central PMCID: 3863629. 23258413 PMC3863629

[6] Yoneyama M, Kikuchi M, Natsukawa T, Shinobu N, Imaizumi T, Miyagishi M, et al The RNA helicase RIG-I has an essential function in double-stranded RNA-induced innate antiviral responses. Nature immunology. 2004 7;5(7):730–7. 10.1038/ni1087 . 15208624

[7] Yoneyama M, Kikuchi M, Matsumoto K, Imaizumi T, Miyagishi M, Taira K, et al Shared and unique functions of the DExD/H-box helicases RIG-I, MDA5, and LGP2 in antiviral innate immunity. Journal of immunology. 2005 9 1;175(5):2851–8. . 16116171 10.4049/jimmunol.175.5.2851

[8] Seth RB, Sun L, Ea CK, Chen ZJ. Identification and characterization of MAVS, a mitochondrial antiviral signaling protein that activates NF-kappaB and IRF 3. Cell. 2005 9 9;122(5):669–82. 10.1016/j.cell.2005.08.012 . 16125763

[9] Kumar H, Kawai T, Kato H, Sato S, Takahashi K, Coban C, et al Essential role of IPS-1 in innate immune responses against RNA viruses. The Journal of experimental medicine. 2006 7 10;203(7):1795–803. 10.1084/jem.20060792 . Pubmed Central PMCID: 2118350. 16785313 PMC2118350

[10] Xu LG, Wang YY, Han KJ, Li LY, Zhai Z, Shu HB. VISA is an adapter protein required for virus-triggered IFN-beta signaling. Molecular cell. 2005 9 16;19(6):727–40. 10.1016/j.molcel.2005.08.014 . 16153868

[11] Liu S, Cai X, Wu J, Cong Q, Chen X, Li T, et al Phosphorylation of innate immune adaptor proteins MAVS, STING, and TRIF induces IRF3 activation. Science. 2015 3 13;347(6227):aaa2630 10.1126/science.aaa2630 . 25636800

[12] Lee RC, Ambros V. An extensive class of small RNAs in Caenorhabditis elegans. Science. 2001 10 26;294(5543):862–4. 10.1126/science.1065329 . 11679672

[13] Lau NC, Lim LP, Weinstein EG, Bartel DP. An abundant class of tiny RNAs with probable regulatory roles in Caenorhabditis elegans. Science. 2001 10 26;294(5543):858–62. 10.1126/science.1065062 . 11679671

[14] Lagos-Quintana M, Rauhut R, Lendeckel W, Tuschl T. Identification of novel genes coding for small expressed RNAs. Science. 2001 10 26;294(5543):853–8. 10.1126/science.1064921 . 11679670

[15] Guo H, Ingolia NT, Weissman JS, Bartel DP. Mammalian microRNAs predominantly act to decrease target mRNA levels. Nature. 2010 8 12;466(7308):835–40. 10.1038/nature09267 . Pubmed Central PMCID: 2990499. 20703300 PMC2990499

[16] Chen K, Rajewsky N. The evolution of gene regulation by transcription factors and microRNAs. Nature reviews Genetics. 2007 2;8(2):93–103. 10.1038/nrg1990 . 17230196

[17] Bogerd HP, Whisnant AW, Kennedy EM, Flores O, Cullen BR. Derivation and characterization of Dicer- and microRNA-deficient human cells. Rna. 2014 6;20(6):923–37. 10.1261/rna.044545.114 . Pubmed Central PMCID: 4024645. 24757167 PMC4024645

[18] Aguado LC, Schmid S, Sachs D, Shim JV, Lim JK, tenOever BR. microRNA Function Is Limited to Cytokine Control in the Acute Response to Virus Infection. Cell host & microbe. 2015 12 9;18(6):714–22. 10.1016/j.chom.2015.11.003 . Pubmed Central PMCID: 4683400. 26651947 PMC4683400

[19] Seo GJ, Kincaid RP, Phanaksri T, Burke JM, Pare JM, Cox JE, et al Reciprocal inhibition between intracellular antiviral signaling and the RNAi machinery in mammalian cells. Cell host & microbe. 2013 10 16;14(4):435–45. 10.1016/j.chom.2013.09.002 . Pubmed Central PMCID: 3837626. 24075860 PMC3837626

[20] Song K, Han C, Dash S, Balart LA, Wu T. MiR-122 in hepatitis B virus and hepatitis C virus dual infection. World journal of hepatology. 2015 3 27;7(3):498–506. 10.4254/wjh.v7.i3.498 . Pubmed Central PMCID: 4381172. 25848473 PMC4381172

[21] Li Y, Shi X. MicroRNAs in the regulation of TLR and RIG-I pathways. Cellular & molecular immunology. 2013 1;10(1):65–71. 10.1038/cmi.2012.55 . Pubmed Central PMCID: 4003181. 23262976 PMC4003181

[22] Androulidaki A, Iliopoulos D, Arranz A, Doxaki C, Schworer S, Zacharioudaki V, et al The kinase Akt1 controls macrophage response to lipopolysaccharide by regulating microRNAs. Immunity. 2009 8 21;31(2):220–31. 10.1016/j.immuni.2009.06.024 . Pubmed Central PMCID: 2865583. 19699171 PMC2865583

[23] Hou J, Wang P, Lin L, Liu X, Ma F, An H, et al MicroRNA-146a feedback inhibits RIG-I-dependent Type I IFN production in macrophages by targeting TRAF6, IRAK1, and IRAK2. Journal of immunology. 2009 8 1;183(3):2150–8. 10.4049/jimmunol.0900707 . 19596990

[24] Meadows SM, Myers CT, Krieg PA. Regulation of endothelial cell development by ETS transcription factors. Seminars in cell & developmental biology. 2011 12;22(9):976–84. 10.1016/j.semcdb.2011.09.009 . Pubmed Central PMCID: 3263765. 21945894 PMC3263765

[25] You F, Wang P, Yang L, Yang G, Zhao YO, Qian F, et al ELF4 is critical for induction of type I interferon and the host antiviral response. Nature immunology. 2013 12;14(12):1237–46. 10.1038/ni.2756 . Pubmed Central PMCID: 3939855. 24185615 PMC3939855

[26] Quinn SR, Mangan NE, Caffrey BE, Gantier MP, Williams BR, Hertzog PJ, et al The role of Ets2 transcription factor in the induction of microRNA-155 (miR-155) by lipopolysaccharide and its targeting by interleukin-10. The Journal of biological chemistry. 2014 2 14;289(7):4316–25. 10.1074/jbc.M113.522730 . Pubmed Central PMCID: 3924294. 24362029 PMC3924294

[27] Yang JK, Yang JP, Tong J, Jing SY, Fan B, Wang F, et al Exosomal miR-221 targets DNM3 to induce tumor progression and temozolomide resistance in glioma. Journal of neuro-oncology. 2017 1;131(2):255–65. 10.1007/s11060-016-2308-5 . 27837435

[28] Lacorazza HD, Miyazaki Y, Di Cristofano A, Deblasio A, Hedvat C, Zhang J, et al The ETS protein MEF plays a critical role in perforin gene expression and the development of natural killer and NK-T cells. Immunity. 2002 10;17(4):437–49. . 12387738 10.1016/s1074-7613(02)00422-3

[29] Chen H, Sun H, You F, Sun W, Zhou X, Chen L, et al Activation of STAT6 by STING is critical for antiviral innate immunity. Cell. 2011 10 14;147(2):436–46. 10.1016/j.cell.2011.09.022 . 22000020

[30] Marsico A, Huska MR, Lasserre J, Hu H, Vucicevic D, Musahl A, et al PROmiRNA: a new miRNA promoter recognition method uncovers the complex regulation of intronic miRNAs. Genome biology. 2013 8 16;14(8):R84 10.1186/gb-2013-14-8-r84 . Pubmed Central PMCID: 4053815. 23958307 PMC4053815

[31] Sedger LM. microRNA control of interferons and interferon induced anti-viral activity. Molecular immunology. 2013 12;56(4):781–93. 10.1016/j.molimm.2013.07.009 . 23962477

[32] Harris TA, Yamakuchi M, Kondo M, Oettgen P, Lowenstein CJ. Ets-1 and Ets-2 regulate the expression of microRNA-126 in endothelial cells. Arteriosclerosis, thrombosis, and vascular biology. 2010 10;30(10):1990–7. 10.1161/ATVBAHA.110.211706 . Pubmed Central PMCID: 3121560. 20671229 PMC3121560

[33] Lodge R, Ferreira Barbosa JA, Lombard-Vadnais F, Gilmore JC, Deshiere A, Gosselin A, et al Host MicroRNAs-221 and -222 Inhibit HIV-1 Entry in Macrophages by Targeting the CD4 Viral Receptor. Cell reports. 2017 10 3;21(1):141–53. 10.1016/j.celrep.2017.09.030 . 28978468

[34] Ishikawa H, Barber GN. STING is an endoplasmic reticulum adaptor that facilitates innate immune signalling. Nature. 2008 10 2;455(7213):674–8. 10.1038/nature07317 . Pubmed Central PMCID: 2804933. 18724357 PMC2804933

[35] Kuleshov MV, Jones MR, Rouillard AD, Fernandez NF, Duan Q, Wang Z, et al Enrichr: a comprehensive gene set enrichment analysis web server 2016 update. Nucleic acids research. 2016 7 8;44(W1):W90–7. 10.1093/nar/gkw377 . Pubmed Central PMCID: 4987924. 27141961 PMC4987924

[36] Dunn GP, Bruce AT, Sheehan KC, Shankaran V, Uppaluri R, Bui JD, et al A critical function for type I interferons in cancer immunoediting. Nature immunology. 2005 7;6(7):722–9. 10.1038/ni1213 . 15951814

[37] Garofalo M, Quintavalle C, Romano G, Croce CM, Condorelli G. miR221/222 in cancer: their role in tumor progression and response to therapy. Current molecular medicine. 2012 1;12(1):27–33. . Pubmed Central PMCID: 3673714. 22082479 10.2174/156652412798376170 PMC3673714

[38] Di Domizio J, Cao W. Fueling autoimmunity: type I interferon in autoimmune diseases. Expert review of clinical immunology. 2013 3;9(3):201–10. 10.1586/eci.12.106 . Pubmed Central PMCID: 3873736. 23445195 PMC3873736

[39] Zitvogel L, Galluzzi L, Kepp O, Smyth MJ, Kroemer G. Type I interferons in anticancer immunity. Nature reviews Immunology. 2015 7;15(7):405–14. 10.1038/nri3845 . 26027717

