# Supplementary figures and images for: MiR-221 negatively regulates innate anti-viral response
Source: PLoS One. 2018 Aug 8;13(8):e0200385. doi: 10.1371/journal.pone.0200385 (PMC6082502; doi:10.1371/journal.pone.0200385)

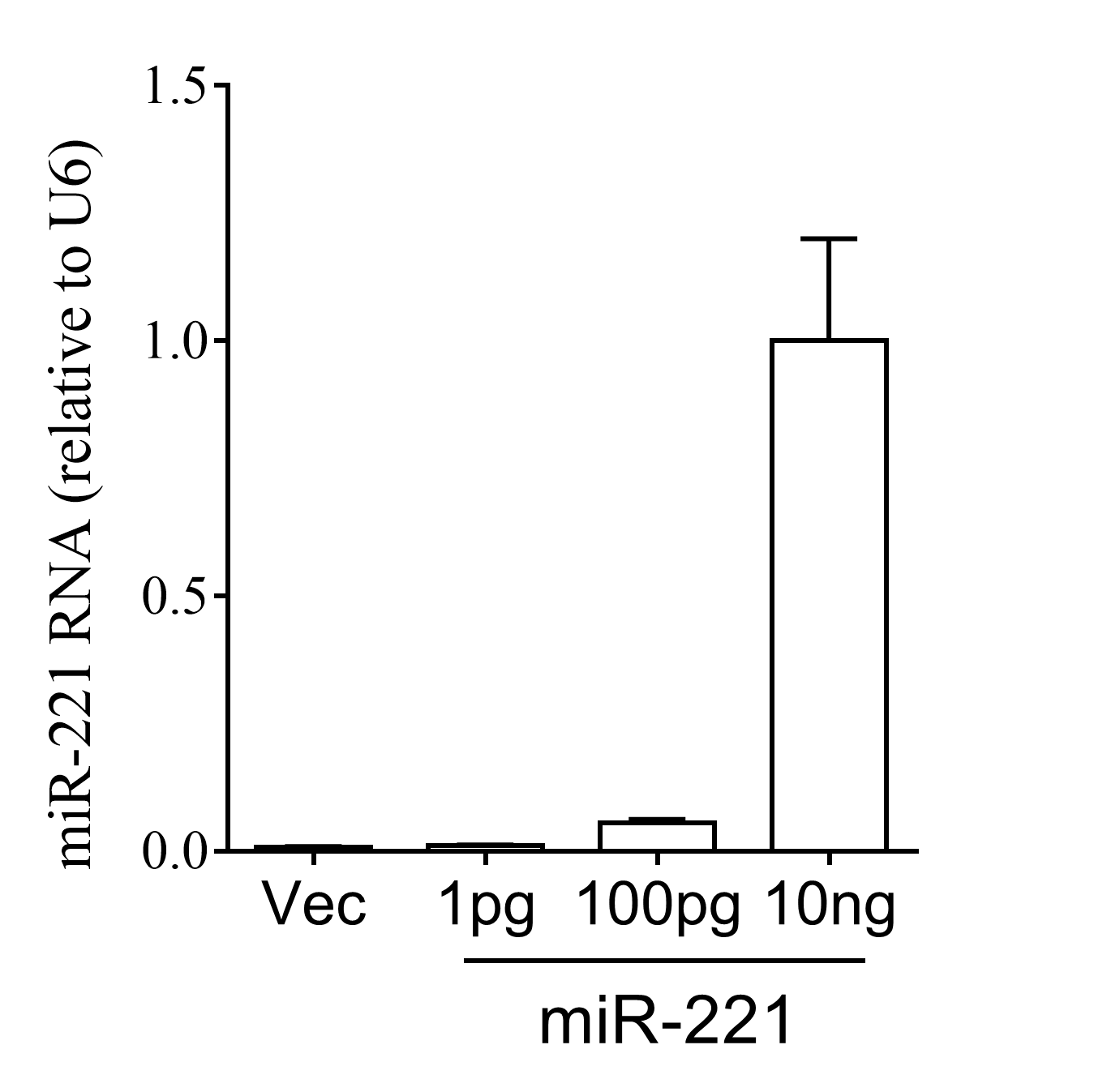

Supplement: S1 Fig — HEK 293T cells were transfected with increasing amount of miR-221 plasmid, 24 hours later, miR-221 transcripts were determined by qPCR. The data are expressed as the mean ± SEM of 2 independent experiments. (n = 2 biological replicates). (TIF) [file pone.0200385.s001.tif]

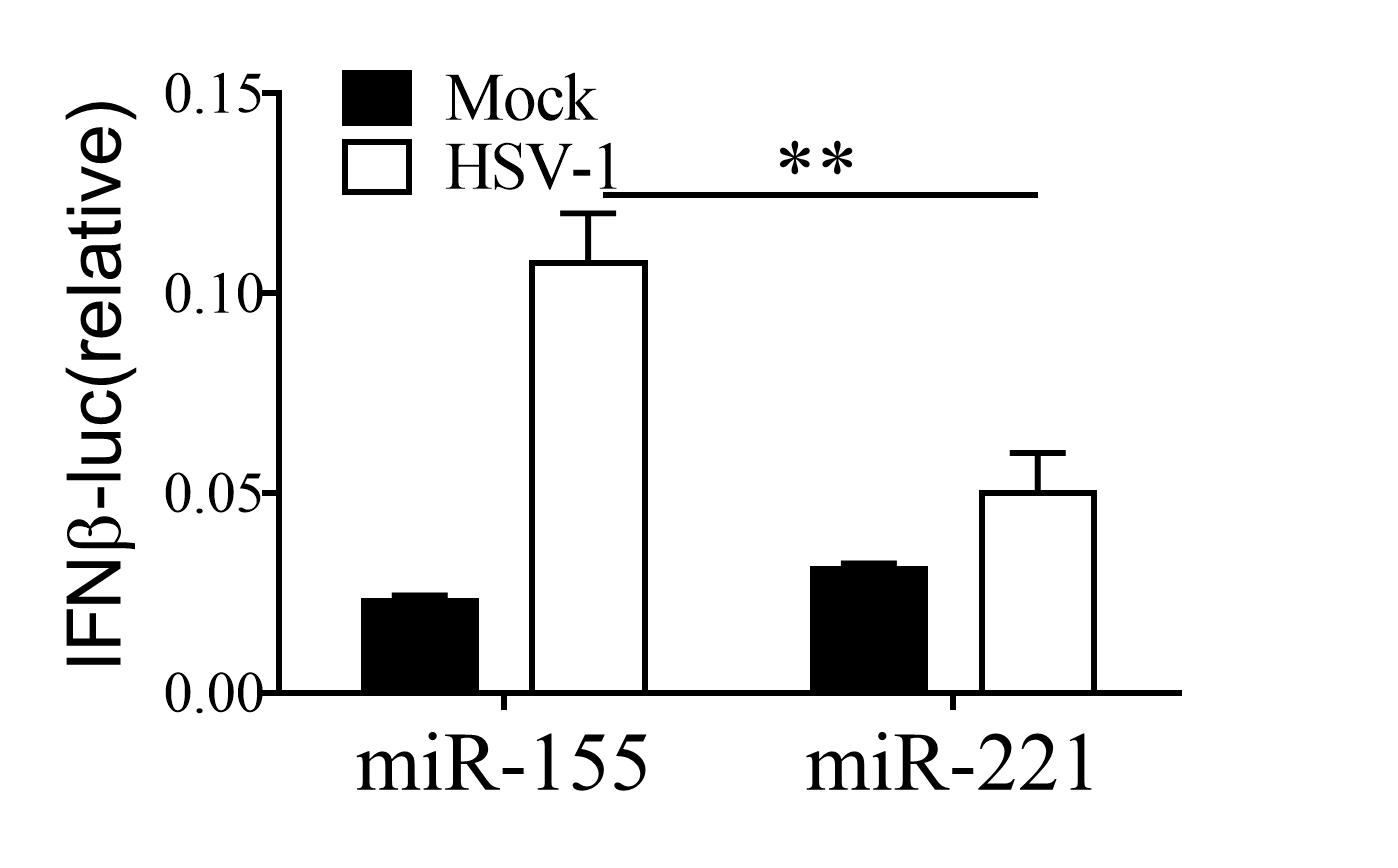

Supplement: S2 Fig — HEK 293T cells were transfected with an IFNβ-Luc plasmid and the indicated plasmids, 12 hours later, the cells were infected with HSV-1. Data were pooled from three independent experiments. The data are expressed as the mean ± SEM of 2 independent experiments. (n = 2 biological replicates). (TIF) [file pone.0200385.s002.tif]

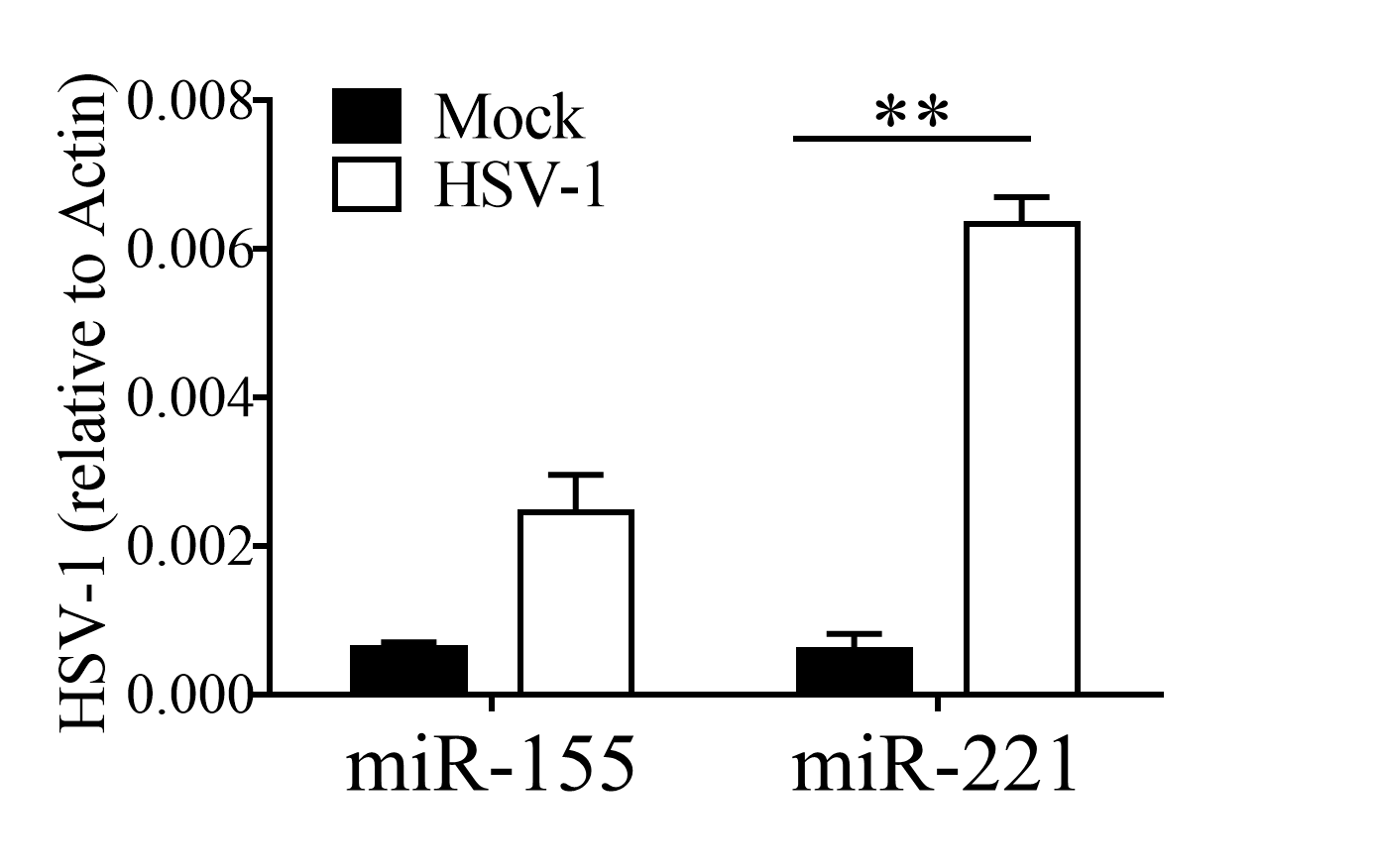

Supplement: S3 Fig — HEK 293T cells were transfected with the indicated plasmids. 12 hours later, the cells were infected with HSV-1. 24 hours later the viral load was measured by qRT-PCR. The data are expressed as the mean ± SEM of 2 independent experiments. (n = 2 biological replicates). (TIF) [file pone.0200385.s003.tif]
